# Case Report: A New Geographic Area for the Presence of the Zoonotic Ocular Nematode, *Onchocerca lupi* in Romania

**DOI:** 10.3389/fvets.2022.941303

**Published:** 2022-07-08

**Authors:** Georgiana Deak, Serenela Toader, Diana Gabriela Soare, Angela Monica Ionică, Marian Taulescu, Andrei Daniel Mihalca

**Affiliations:** ^1^Department of Parasitology and Parasitic Diseases, Faculty of Veterinary Medicine, University of Agricultural Sciences and Veterinary Medicine of Cluj-Napoca, Cluj-Napoca, Romania; ^2^Certovet, Veterinary Clinic of Năvodari, Năvodari, România; ^3^Histovet, Veterinary Laboratory of Bucharest, Bucureşti, România; ^4^Molecular Biology and Veterinary Parasitology Unit (CDS 9), “Regele Mihai I al României” Life Science Institute, University of Agricultural Sciences and Veterinary Medicine of Cluj-Napoca, Cluj-Napoca, Romania; ^5^Molecular Diagnosis Laboratory, Clinical Hospital of Infectious Diseases of Cluj-Napoca, Cluj-Napoca, Romania; ^6^Department of Pathology and Parasitic Diseases, Faculty of Veterinary Medicine, University of Agricultural Sciences and Veterinary Medicine of Cluj-Napoca, Cluj-Napoca, Romania

**Keywords:** *Onchocerca lupi*, Romania, zoonosis, dogs, new location

## Abstract

**Introduction:**

*Onchocerca lupi* is a zoonotic parasite of carnivores reported in countries from Europe, North America, Asia and Africa. In Romania, canine ocular onchocercosis was first reported in dogs in 2016 from Târgovişte (Dâmboviţa County) and Oneşti (Bacău County) and more recently, it was detected in an adult stray dog exported to UK from an unspecified location in Romania.

**Methods:**

A 4-years old male mongrel dog was referred to a private veterinary clinic in Năvodari (Constanţa County), Romania due to ocular disorders. The dog was originally from a public dog shelter in Balş (Olt County) and transferred in June 2020 to a dog shelter in Medgidia (Constanţa County). A cytological examination followed by surgical removal of the granuloma localized at the internal angle of the right eye, and molecular identification of the extracted nematode were done.

**Results:**

The cytological examination confirmed a parasitic granuloma. Skin biopsy and PCR confirmed the infection with *O. lupi*. Two doses of moxidectin and imidacloprid were administered after the surgery. No relapse observed after a period of follo-up of 11 months.

**Conclusion:**

*Onchocerca lupi* is present in dogs from Romania with its actual distribution remaining unknown.

## Introduction

Canine ocular onchocercosis is produced by *Onchocerca lupi*, a zoonotic filarioid parasite of carnivores, having a wide geographical distribution (1, 2). Since its original description in a wolf (*Canis lupus*) from Georgia (3), the presence of *O. lupi* was detected again after more than 30 years, in dogs from Hungary (4). The currently known distribution includes countries from Europe, North America, Asia and Africa, where domestic dogs, cats and humans act as definitive hosts (2). In Romania, canine ocular onchocercosis was first reported in dogs from Târgovişte (Dâmboviţa County) and Oneşti (Bacău County) (5) and more recently, it was detected in an adult stray dog exported to the UK from an unspecified location in Romania (6). *Onchocerca lupi* has an indirect, but incompletely known life-cycle with carnivores acting as definitive hosts. However, intermediate hosts are still unknown, but are probably represented by hematophagous arthropods (2, 7). Adult worms typically reside in the ocular connective tissue (conjunctiva, subconjunctiva, eyelids, nictating membrane). The larvae are localized in the subcutaneous tissue (8). Occasionally, aberrant localizations of adults in the larynx of dogs (9) or spinal cord of humans (10) have been reported.

The clinical disease caused by *O. lupi* in dogs includes a wide variety of manifestations, such as acute or chronic ocular infections with periorbital swellings, epiphora, or photophobia which could eventually cause blindness if left untreated (2, 11, 12). Infection may also be asymptomatic, but these cases are rarely diagnosed (2). In aberrant localizations in soft tissues of the larynx, stenosis of the glottal and tracheal ducts with dyspnea and cyanosis were observed (9). Infected humans most frequently develop ocular problems and subcutaneous granulomatous cysts. The aberrant localization of the adult nematodes in the spinal cord was associated with compression and headaches (13, 14).

Diagnosis of canine onchocercosis is based on the presence of ocular nodules during ophthalmological examinations, or by more invasive methods such as skin-snip biopsies followed by morphological and molecular identification of microfilariae (8). More recently, a less invasive method, suitable also for large scale epidemiological studies, consisting in the serological detection of antigens was developed (15).

The most common treatment is the surgical excision of the nodules containing the adult nematodes followed by the administration of macrocyclic lactones and antibiotics. Up to now, there are no labeled products against canine ocular onchocercosis and the knowledge about the treatment protocols is very limited, which urges the need of further studies on this topic (2).

Considering the severity of the disease and the zoonotic character of *O. lupi*, the aim of the present paper was to highlight the existence of a possible new risk area in Romania and raise further awareness among veterinary clinicians to include canine onchocercosis as a differential diagnosis of ocular disorders.

## Materials and Methods

The dog was originally from a public dog shelter in southern Romania, Balş (Olt County), in the southern region of Romania (44.34N, 24.09E) at a low altitude (132 m a.s.l.) with a continental-temperate climate^1^. In June 2020, the animal was transferred to a dog shelter in Medgidia (Constanţa County, south-east Romania) (44.24N, 28.29E). Medgidia is situated at 75 m altitude a.s.l. and is localized between the Danube river (in the west) and the Black Sea (in the east). This locality is characterized by very hot summers and short cold winters, with a continental-temperate climate^1^. Six months later, in December 2020, an animal attendant reported the presence of a nodule at the internal angle of the right eye. In February 2021, the 4-years old male mongrel dog was referred to a private veterinary clinic in Năvodari (Constanţa County), Romania due to ophthalmological problems. Additionally, an ocular ultrasound was performed.

The dog received local treatment with 1% prednisolone eye drops for 10 consecutive days and an oral systemic treatment with tablets, 10 mg/day prednisolone (Prednicortone, Dechra) for 7 consecutive days. Additionally, the dog received a first dose of oral milbemycin oxime + praziquantel (Milprazon, KRKA), according to the label, followed by another similar dose after 10 days administered by the veterinarian.

The clinical evolution of the dog was followed by the shelter workers. However, due to the persistence of the ocular nodule, 3 months after the initial treatment, the dog was referred again to the same private clinic (May 2021). A fine-needle aspiration from the conjunctival mass was done, followed by cytological examination using Diff Quick stain.

**Figure 1:**
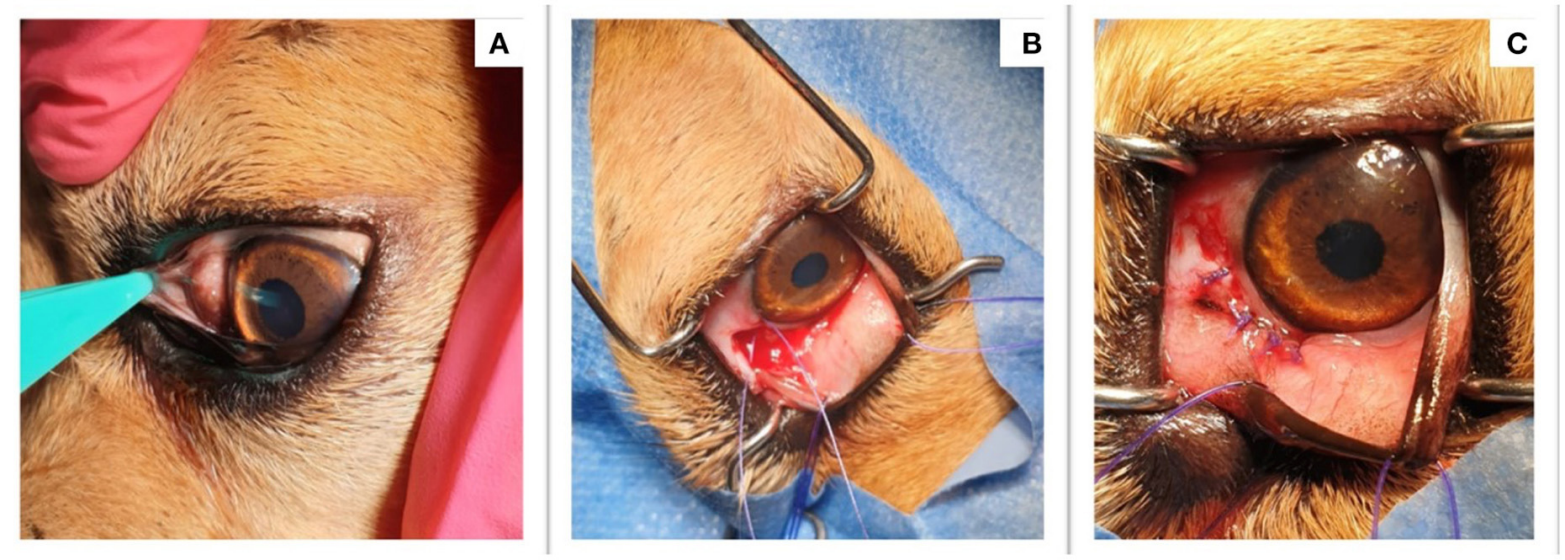
Clinical aspect before and after the surgical removal of the nodule. **(A)** The presence of a conjunctival nodule; **(B)** The nodule was excised during surgery; **(C)** The aspect of the dogs' eye after the nodule was removed.

After the cytopathological diagnosis, the nodule was surgically removed (Figures 1B,C), following general anesthesia (ketamine and acepromazine). The excised granuloma was cut into two parts and placed in two tubes containing 10% buffered formalin for histopathological examination and absolute ethanol, for the molecular analysis of the parasite, respectively. During the surgery, a small skin-snip biopsy was also collected from the interscapular area and analyzed for the presence of microfilariae and stained with methylene-blue as previously described (8). In addition, a blood sample was collected for the detection of microfilaria of other filarioids.

For histological examination, fixed conjunctival and subconjunctival tissues with nodular lesions were paraffin-embedded. Ten serial 3 μm-sections were cut and processed for routine hematoxylin and eosin (HE) staining.

Genomic DNA was isolated from an approximately 2 cm long fragment of nematode recovered from the nodule and from 200 μL of whole blood using a commercially available kit (Isolate II Genomic DNA Kit, Bioline, London, UK), according to the manufacturer's instructions. The nematode fragment was characterized by amplification and sequencing of a ~670 bp fragment of the *cytochrome c oxidase* subunit I (*cox*1) gene, using universal Spirurid primers (NTF, NTR), as previously described (16). The sequence was analyzed by Basic Local Alignment Tool (BLAST) analysis. The blood sample was screened for the presence of three species of filarioids (*Dirofilaria immitis, D*. *repens*, and *Acanthocheilonema reconditum*), by means of a multiplex PCR amplifying partial regions of the *cytochrome c oxidase* subunit 1 (*cox*1) gene, as described in the literature (17).

The dog received 2 spot-on doses of moxidectin and imidacloprid (Advocate, Elanco), 1 month apart. A second skin biopsy was performed 4 months after the surgery (October 2021). The dog was continuously followed by visual inspection by the dog keepers and regulated check-ups at the private clinic.

## Results

The clinical examination of the dog revealed a good general condition apart from the subconjunctival nodule on the surface of the sclera at the internal angle of the right eye (Figure 1A). A more thorough ophthalmological examination exposed mild conjunctivitis, normal photo-pupillary reflexes, and normal eye pressure (left eye-OS: 19 mmHg; right eye-OD: 18 mmHg; normal values: 15–20 mmHg). The ocular ultrasound revealed ocular globe diameters of 1.73 cm for OS and 1.78 for OD. The clinical examination suggested a differential diagnosis of parasitic conjunctival granuloma, nodular episcleritis, or scleral tumor.

**Figure 2:**
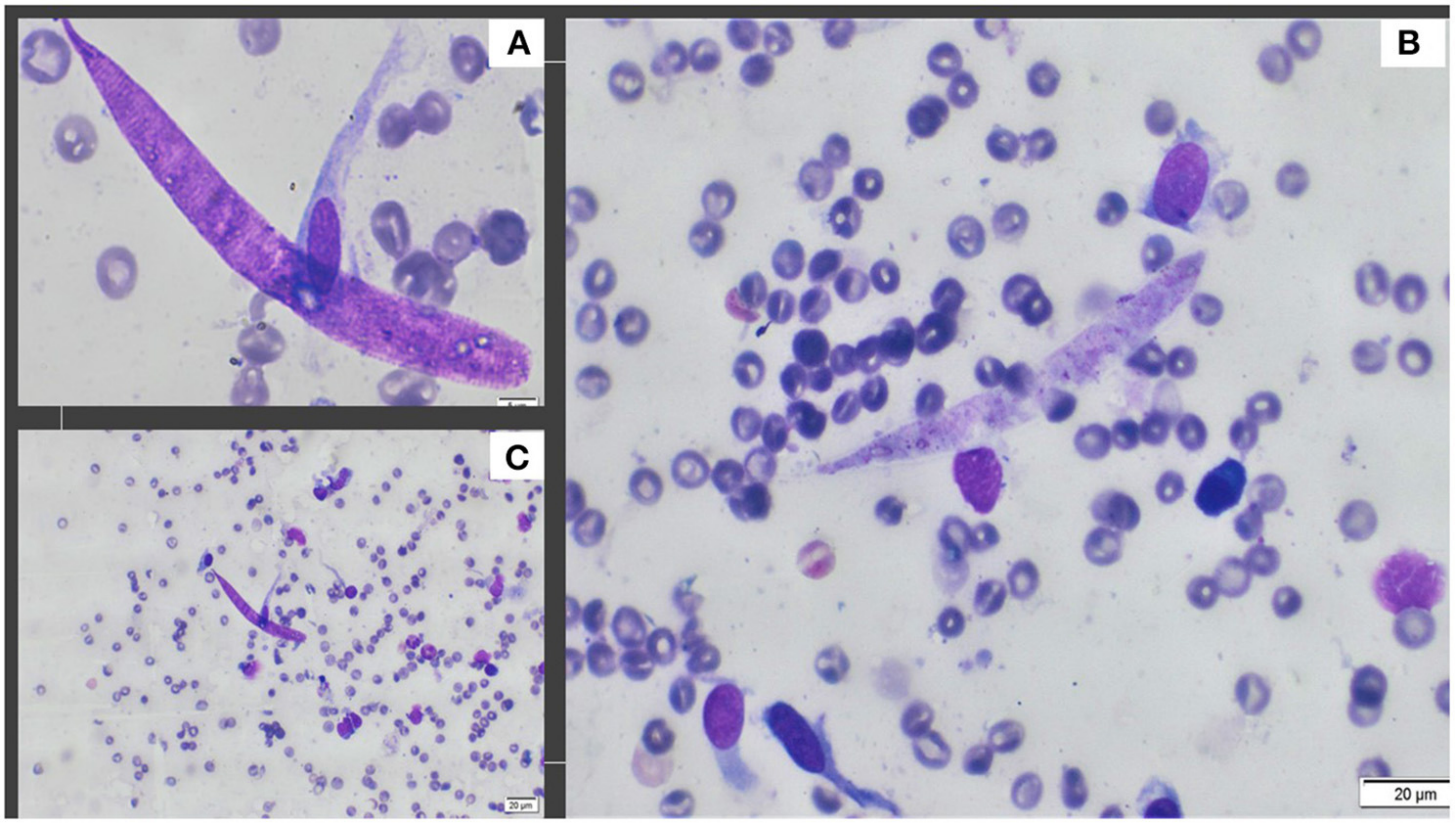
Photographs obtained from cytology. **(A)** The detailed aspect of *Onchocerca lupi* larva obtained with 100X; **(B)** A typical larva and presence of fibroblasts, endothelial cells and plasma cells, 40X; **(C)** Presence of larva and plasma cells, eosinophils, fibroblasts and non-keratinized epithelial cells, 20X.

The examination of the aspirate revealed the presence of macrophages, epithelioid cells, a few multinucleated giant cells, reactive fibroblasts, plasma cells, small lymphocytes, and a few squamous epithelial cells. The presence of larval nematodes (ca. 105 μm in length and 7–8 μm in width) concluded the diagnosis of a parasitic granuloma (Figure 2).

**Figure 3:**
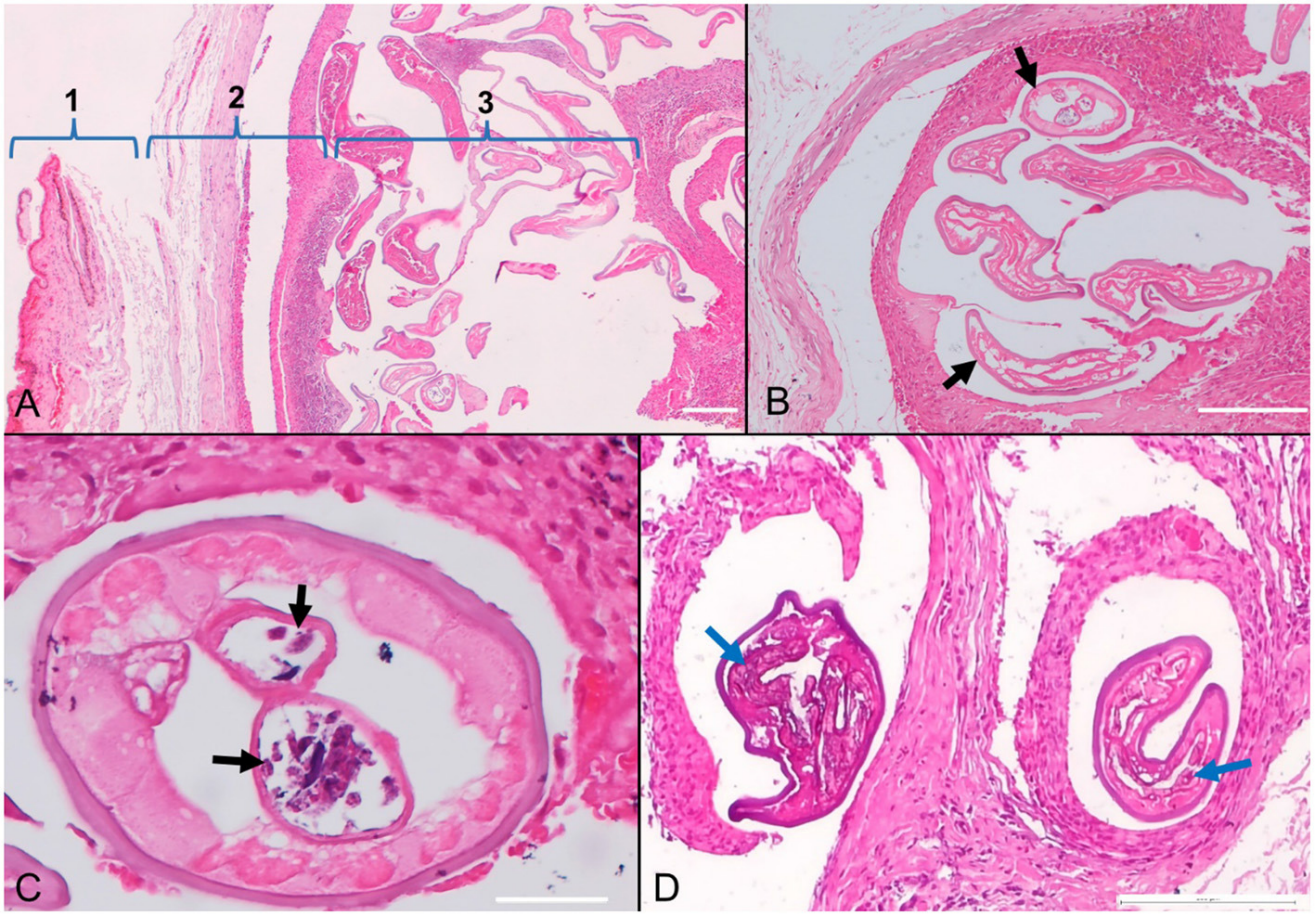
Photomicrographs of the conjunctiva in a dog (*Canis familiaris*) with ocular onchocercosis. **(A)** The subconjunctival tissue (1-conjunctiva) is expanded by a granulomatous inflammatory reaction and fibrosis (2) centered on numerous filarial nematodes (3). **(B)** Subconjunctival granuloma containing parasites (black arrows). **(C)** The nematode cross-section shows paired uteri with microfilariae (black arrows). **(D)** Granulomatous nodules with degenerated and mineralized parasites (blue arrows); HE stain.

The subconjunctival tissue was markedly expanded by numerous, variably sized, well delimited, and encapsulated inflammatory nodules (Figures 3A,B). They were composed mainly of epithelioid macrophages and multinucleated giant cells (both foreign body and Langhans types) (granulomas) with fewer small lymphocytes, plasma cells, eosinophils, and neutrophils. At the periphery, the granulomas were enclosed by a moderate amount of fibrous tissue. Each nodule contained 1-6 filarial nematodes (Figures 3A,B), with an individual diameter varying from 100 to 150 μm and showed a thick eosinophilic cuticle with cuticular ridges, very thin layer of atrophied coelomyarian muscle which was multifocally replaced by hypodermis, and a pseudocoelom containing paired uteri and a small intestinal tract. The uterus occasionally contained cross-sections of microfilariae (Figure 3C). Some nematodes showed a basophilic granular material (dystrophic mineralization) (Figure 3D).

The sequence had a 100% nucleotide identity to seven *O. lupi* isolates [genotype 1–according to (2)]: six from USA (MW577256, JX080028-JX080031, NC_056960), and one from the dog imported from Romania into the UK (MW835251). The sequence was deposited in GenBank under the Accession Number ON319015. The blood samples was negative for microfilariae.

**Figure 4:**
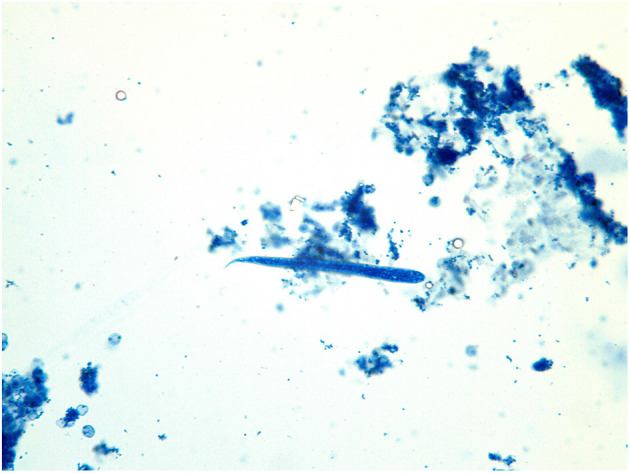
*Onchocerca lupi* larva recovered from the skin-snip biopsy.

The analyzed skin snip revealed the presence of one larva (Figure 4) which was compatible with the morphology of *O. lupi* larvae (11, 18). The detected microfilaria showed a rounded head with a small tooth in the cephalic area and a flattened body with a short tail. The larva was 109 μm long and 6.9 μm wide. The Knott's test and the PCR from the blood were both negative for other filariae. The second skin biopsy performed 4 months after the surgery was negative. No clinical relapses were observed till the present (i.e., 11 months later).

## Discussion

The present clinical case provides new information on the spread of zoonotic *O. lupi* in Romania and highlights the need for further surveillance to better define the risk areas. In our case, a topic of debate is the actual geographic site of infection of the dog, which was moved from Olt to Constanţa County at the beginning of the summer (June 2020), 6 months before the nodule became visible (December 2020), and eleven months before the larvae were detected in the skin (May 2021). However, the actual date of positivation of skin for microfilariae could not be estimated, as no previous biopsies were done.

The incubation period for *O. lupi* is not known, but some reports in humans suggest it to be even as short as 30 days (19). However, as for other *Onchocerca* species, it was suggested that it can be up to 18 months or more (1). Similar scarce data exist on the prepatent period which is estimated to be several months with the patency lasting for several years (11, 20).

**Figure 5:**
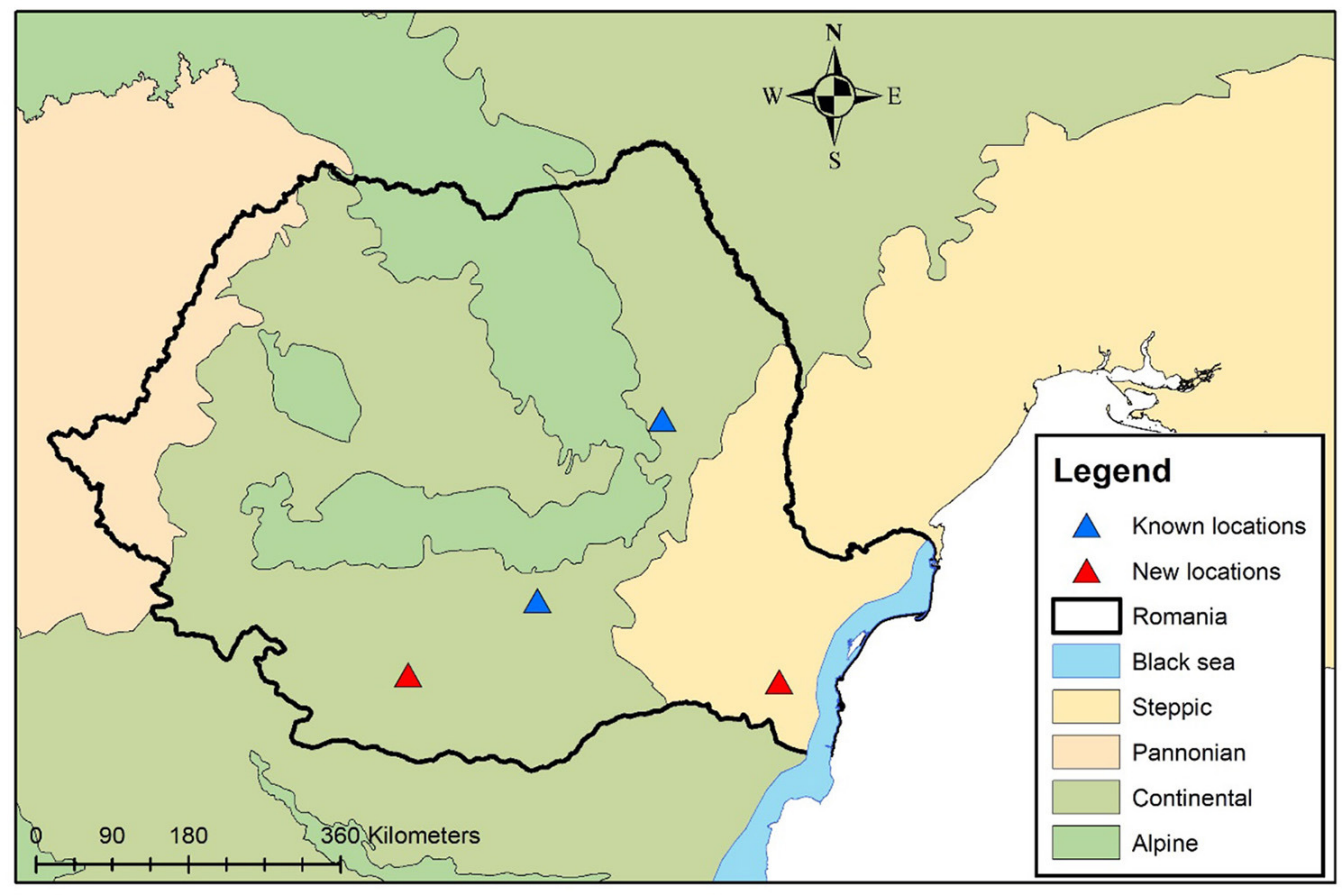
Geographical distribution of *O. lupi* in domestic dogs from Romania. Blue triangles show previously reported cases. The red triangles show the two new possible locations.

Considering all this data, the exact location in which the dog got infected is unknown and could be represented by any of the two locations (Balş - Olt County or Medgidia, Constanţa County, the latter being a highly touristic area during the summer due to the proximity to the Black Sea). In both two counties, the presence of the assumed to be vectors (family Simuliidae) was reported (21) and the climatic conditions are also favorable for their development. In any of the cases, the location would represent a new locality for Romania (Figure 5).

The most common treatment protocol is the surgical removal of the nematodes, followed by microfilaricidal drugs. Ivermectin is known to stop microfilariae production and to sterilize females; however, this seems to work only for the European strain as in the US more than half of the dogs treated with ivermectin presented recurrence of the clinical signs (12, 14, 22). In a more recent publication, a relapse of new worms after the surgical extraction of the nodules was reported, accompanied by severe lesions, incompatible with saving the ocular globe (6). Relapses were previously mentioned, but they were observed in the healthy eye supporting the existence of undiagnosed nematodes, before forming nodules (23). Such cases of subclinical infection, together with wild canids, serve as reservoirs of infection for healthy animals and also for humans. Even though the complete life cycle of *O. lupi* is still unknown, the possible arthropod vector is considered to be represented by Simuliidae flies, which are known to be present in Romania (2, 24).

Movements of the dogs from one location to another, together with their owners or in dog shelters, raise the risk of spreading diseases. Most of the dogs from private dog shelters in Romania are exported to more developed European countries. Unfortunately, rehoming the dogs in such a manner increases the risk of spreading diseases, including parasites, a well-known phenomenon for dogs exported from Romania (6, 25).

Moreover, the zoonotic risk is highlighted by the presence of the infected dogs together with the presence of the potential vectors in both mentioned locations. The disease in humans can be very serious in some cases (13, 14), and it is a reason for advocating the importance of updating the currently known distribution in Romania and to further investigate the vectors.

Research on this topic is encouraged as there are still unsolved problems, namely the identity of the arthropod vector, together with the development of a less-invasive diagnostic tools, which could be used for large-scale surveys.

## Conclusion

Infections with *Onchocerca lupi* in dogs are present in Romania and the actual distribution of this nematode remains unknown. Many dogs could be sub-clinically infected, and the actual prevalence is very hard to determine by the use of skin-snip biopsies.

## Data Availability Statement

The datasets presented in this study can be found in online repositories. The names of the repository/repositories and accession number(s) can be found in the article/supplementary material.

## Ethics Statement

Ethical review and approval was not required for the animal study because it was not necessary as the used protocol did not imply any procedures which ask for ethics approval.

## Author Contributions

GD performed the skin-snip analyses, conceived the study, and wrote the manuscript. ST performed the sample prelevation, did the surgery, and was responsible for the follow-up. DS performed the cytological examination. AI performed the molecular work and revised the manuscript. MT performed the histological diagnosis. AM coordinated the study and critically revised the manuscript. All authors contributed to the article and approved the submitted version.

## Conflict of Interest

The authors declare that the research was conducted in the absence of any commercial or financial relationships that could be construed as a potential conflict of interest.

## Publisher's Note

All claims expressed in this article are solely those of the authors and do not necessarily represent those of their affiliated organizations, or those of the publisher, the editors and the reviewers. Any product that may be evaluated in this article, or claim that may be made by its manufacturer, is not guaranteed or endorsed by the publisher.

## Footnotes

^1^Available online at: https://weatherspark.com/y/90950/Average-Weather-in-Romania-Year-Round
